# Ultrasound-guided greater occipital nerve block for chronic migraine: a systematic review and meta-analysis

**DOI:** 10.1186/s13005-025-00554-1

**Published:** 2025-11-24

**Authors:** Haneen Sabet, Abdallah Abbas, Moaz Elsayed Abouelmagd, Ahmed Samir, Mohamed Mohsen Helal, Mohamed El-Moslemani, Ahmed F. Younis, Obai Yousef, Rovan Ahmed Rouby, Alaa Abd-Elsayed

**Affiliations:** 1 Faculty of Medicine, South Valley University, Qena, Egypt; 2 Faculty of Medicine, Al-Azhar University, Damietta, Egypt; 3 Faculty of Medicine, Cairo University, Cairo, Egypt; 4 Faculty of Medicine, Zagazig University, Zagazig, Egypt; 5 Department of Neurosurgery, Tishreen University Hospital, Latakia, Syria; 6 Faculty of Medicine, Fayoum University, Fayoum, Egypt; 7 Department of Anesthesiology and Pain Management, University of Wisconsin School of Medicine and Public Health, Madison, WI USA

**Keywords:** Greater occipital nerve block, Pulsed radiofrequency, Sphenopalatine ganglion block, Chronic migraine

## Abstract

**Objective:**

This systematic review and meta-analysis evaluated the safety and efficacy of ultrasound-guided greater occipital nerve block (US-GONB) in managing chronic migraine, comparing outcomes with sham, pulsed radiofrequency (PRF), sphenopalatine ganglion block (SPG), and combination interventions.

**Methods:**

Following PRISMA guidelines, a comprehensive search of PubMed, Scopus, Cochrane Library, and Web of Science was conducted until February 5, 2025. Eligible studies included clinical trials and observational studies assessing the efficacy and safety of US-GONB in migraine patients. Two reviewers independently performed data extraction and risk of bias assessment. Meta-analyses utilized RevMan 5.4, reporting mean differences (MD) with 95% confidence intervals (CI), applying random- or fixed-effects models based on heterogeneity.

**Results:**

Six studies (*n* = 344) were included: four randomized controlled trials (RCTs), one non-RCT, and one retrospective cohort. Compared to sham, US-GONB significantly reduced pain intensity (MD: –3.57 points, 95% CI: [–3.95, –3.19]), monthly headache days (MD: –12.12 days, 95% CI: [–13.95, –10.29]), and analgesic use (MD: –2.05 analgesics, 95% CI: [–2.48, –1.62]). Efficacy relative to PRF was comparable; outcomes improved when PRF was added. SPG often showed superior results in head-to-head comparisons. Dizziness was the most frequent adverse event (38.9%), with serious complications being rare.

**Conclusions:**

US-GONB may be a well-tolerated, minimally invasive treatment for chronic migraine, particularly when pharmacologic strategies fail. It offers clinically meaningful improvements over sham but shows variable efficacy against active comparators. Some studies indicate potential synergistic effects with PRF. Limitations include small sample sizes and methodological heterogeneity, warranting larger standardized RCTs to inform clinical guidelines.

**Supplementary Information:**

The online version contains supplementary material available at 10.1186/s13005-025-00554-1.

## Introduction

Migraine is a common neurological disorder that affects individuals across all age groups, with a reported prevalence of approximately 18% in men and up to 43% in women [1–3]. According to the Global Burden of Disease Study 2019, it ranks as the second leading cause of years lived with disability. Chronic migraine, a more persistent and debilitating form, affects about 5% of the general population [4].

Pharmacological management of migraine typically includes nonsteroidal anti-inflammatory drugs (NSAIDs), antiemetics, and triptans [5]. However, several challenges limit the effectiveness of these treatments, including inadequate symptom relief, adverse effects, and contraindications in specific populations, such as pregnant or lactating women [6]. Moreover, a well-recognized complication of pharmacologic management is medication overuse headache, a secondary headache disorder resulting from frequent use of acute migraine medications [7]. The most relevant hallmark of migraine is its considerable heterogeneity in clinical presentation, associated comorbidities, and treatment response, which makes identifying the optimal therapy for each patient challenging [8]. Medication overuse headache is associated with significant impairment in quality of life and highlights the limited long-term efficacy of pharmacological strategies [7].

With the growing need for more sustainable and better-tolerated treatment options, there has been increasing interest in interventional techniques for migraine pain management [9]. Peripheral nerve and ganglion blocks, including greater occipital nerve block (GONB), stellate ganglion block (SGB), sphenopalatine ganglion block (SPG), and cervical ganglion block (CGB), have emerged as promising modalities [9].

These are typically outpatient, minimally invasive procedures designed to interrupt pain signaling, either through pulsed radiofrequency (PRF) or the injection of local anesthetics (like lidocaine) and sometimes steroids near the target nerves. This approach has been successfully used since the 1960 s [10] and is generally well-tolerated with few serious side effects [11]. Among these, GONB is one of the most widely used blocks, and it can be employed as a primary treatment or as a second-line option when other methods fail [10].

More recently, ultrasound (US) guidance has gained traction in chronic pain clinics to improve the accuracy and safety of interventional procedures, including GONB [12]. Studies suggest that ultrasound guidance can improve the precision of nerve localization, thereby enhancing the block's efficacy and safety [13]. For example, a 2011 study reported superior short-term outcomes with US-GONB compared to the traditional landmark-guided approach [14]. Additionally, combining PRF with US-GONB has shown promise in improving immediate and long-term pain relief [13].

Although some meta-analyses have assessed the safety and efficacy of GONB compared to sham or alternative interventions, a focused and comprehensive synthesis of evidence specifically on US-GONB in migraine management is still lacking [15, 16]. This meta-analysis aims to fill this gap by systematically evaluating the safety, efficacy, and clinical outcomes of US-GONB in patients with migraine compared to other interventions.

## Methods

This systematic review and meta-analysis follow the Preferred Reporting Items for Systematic Reviews and Meta-Analyses (PRISMA) guidelines [17].

### Inclusion and exclusion criteria

Eligibility criteria were defined based on the following PICOS framework:

- Population: Patients diagnosed with migraine (acute or chronic, with or without aura).
- Intervention: US-GONB.
- Comparator: PRF, SPG, sham procedures, or other alternative interventions.
- Outcomes: Primary: Pain intensity and monthly headache days. Secondary: headache attack duration (hours), number of analgesic drugs taken per month, and adverse events (AEs).
- Study design: Clinical trials or observational studies.

Any study that did not meet these inclusion criteria was excluded.

### Literature search and study selection

We performed a systematic search across PubMed, Scopus, Cochrane Library, and Web of Science (WOS) up to February 5, 2025, using the following search strategy: ("ultrasound-guided" OR "US-guided" OR "ultrasonography-guided") AND ("nerve block" OR "block*" OR "nerve blockade" OR "stellate ganglion block" OR "greater occipital nerve block" OR "lesser occipital nerve block" OR "sphenopalatine ganglion block" OR "auriculotemporal nerve block" OR "supraorbital nerve block" OR "supratrochlear nerve block" OR "cervical ganglion block") AND ("migraine" OR "chronic migraine" OR "headache" OR "migraine with aura" OR "refractory").

Search results were imported into the Rayyan software, and duplicates were removed [18]. Two independent reviewers conducted screening in two phases: (1) title and abstract screening, followed by (2) full-text screening based on PICOS criteria. Disagreements were resolved by consultation with a third reviewer. Reference lists of included studies were also screened for additional eligible studies.

### Data extraction and bias risk assessment

Following selection, eligible studies were assessed using a predefined spreadsheet for data extraction and bias risk assessment. At least two reviewers independently extracted data and evaluated study bias risk. Extracted data included baseline characteristics, interventions, comparators, outcome measures, and follow-up durations.

Risk of bias for randomized controlled trials (RCTs) was assessed using the Cochrane Risk of Bias 2 (ROB 2) tool, evaluating domains such as randomization, blinding, and outcome reporting [19]. Additionally, for non-randomized studies, the ROBINS-I tool was employed [20]. For observational studies, the Newcastle–Ottawa Scale (NOS) was used, covering selection (max 4 points), comparability (max 2 points), and outcome ascertainment (max 3 points) [21]. A third reviewer resolved any discrepancies in data extraction or bias risk assessment.

### Statistical analysis

Meta-analysis was conducted using Review Manager software (RevMan 5.4) [22]. We used the Meta-Analysis Accelerator tool to calculate baseline changes and convert between various statistical measures [23]. Pooled effect sizes are reported as weighted mean differences (MD) with 95% confidence intervals (CI). Heterogeneity was assessed using the chi-square and *I*^2^ statistics. We considered data significantly heterogenous when (P < 0.1 or I^2^ > 50%). A random-effects model has been applied to account for any heterogeneity and provide more conservative values since it accounts for variability between studies. A leave-one-out sensitivity analysis was performed to identify potential sources of heterogeneity [24].

## Results

### Search strategy and screening

**Fig. 1:**
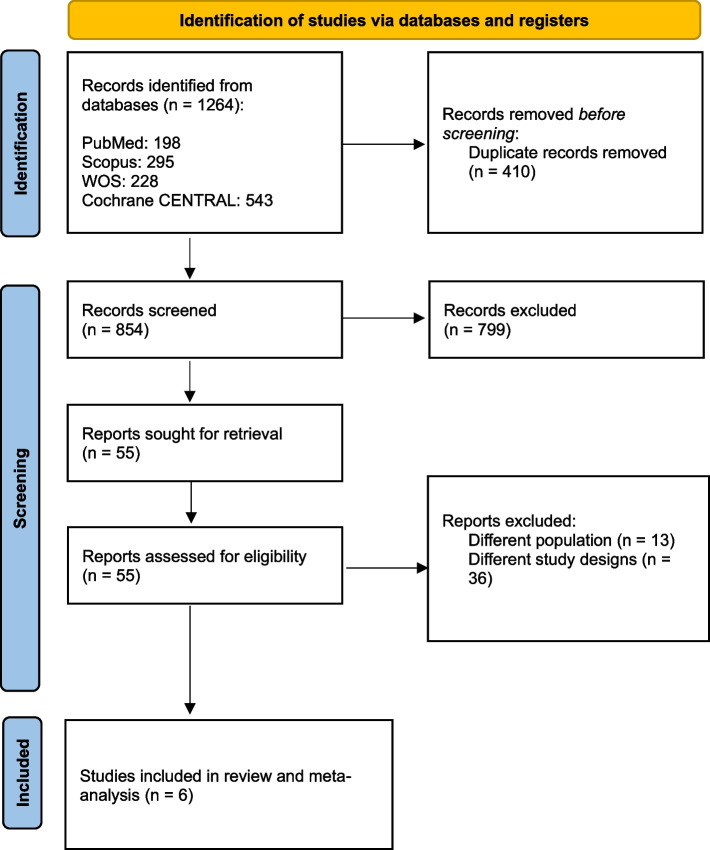
PRISMA flow diagram. The flowchart of the study selection is based on PRISMA guidelines

A comprehensive search across four different databases initially yielded 1264 studies. Following the removal of duplicates, 854 records remained. Screening was conducted in two stages: title and abstract review, followed by full-text assessment, based on the predefined PICO criteria. This process resulted in the inclusion of 6 studies [25–30]. For a detailed selection process overview, please refer to the PRISMA flow diagram (Fig. 1).

### Baseline characteristics

Six studies were included in this analysis. Four were randomized controlled trials (RCTs) [25–28], one was a non-RCT [29], and one was a retrospective cohort [30]. All studies investigated patients with chronic migraine. Comparators included PRF, SPG block, sham treatment, trapezius muscle injection (TPI), and combination interventions.

The total pooled sample across all included studies was 344 patients, with 164 receiving US-GONB (G1) and 180 receiving comparator interventions (combined G2 and G3). Among these, 29 males (17.7%) were in the US-GONB group, and 26 males (14.4%) were in the comparator group. The reported mean age ranged from 25.75 to 44.15 years in the US-GONB group and 27.67 to 47.33 years in the comparator group. Two studies used a single US-GONB protocol, while four employed repeated US-GONB protocols. The anatomical approach was proximal in most studies, with two using a distal approach. The duration of follow-up ranged from one month to six months, with most studies assessing outcomes at one or three months. For additional details on study designs, sample characteristics, and interventions, refer to Tables 1 and 2.

**Table 1:** Summary of the included studies

Study ID	Country	Design	Sample size	Disease studied	Diagnostic criteria	Intervention, frequency (location)	Comparator	Outcomes measured and time	Adjuvant anesthesia protocol	
Taha 2025 [25]	Egypt	RCT	53	Chronic resistant migraine	EHF	Repeated (proximal) US-guided GONB	Repeated SPG blockade and sham	Number of headache days, duration in hours, pain intensity, total Pain Index, number of analgesic tablets, HIT-6 score and MIDAS score in days	NA	3 months
Saracoglu 2024 [26]	Turkey	RCT	32	Chronic migraine	ICHD-3	Single (distal) US-guided GONB	Single PRF + GONB	Frequency and severity of migraine attacks, number of headache days, and number of analgesic tablets	2 mL of 0.25% bupivacaine	6 months
Ertilav 2024 [28]	Turkey	RCT	67	Chronic migraine	ICHD-3	Repeated (distal) US-guided GONB	Single PRF (GON)	Pain severity (VAS), MIDAS, and medication overuse	1% subcutaneous lidocaine, a 21-gauge 5 cm, after negative aspiration, a block was performed with 3 mL of 2% Prilocaine	1 month and 6 months
Balta 2023 [30]	Turkey	Retrospective cohort study	70	Chronic migraine	ICHD-3	Repeated (proximal) US-guided GONB	Repeated SPG blockade	Headache days, responder rate, attack severity, attack frequency, medication overuse at 1 st and 3rd month follow-up	Bupivacaine 0.5% (1.5 cc) was injected into each GON with a 21-gauge 1.5-inch needle once weekly for four sessions	1 month and 3 months
Perdecioglu 2023 [27]	Turkey	RCT	62	Chronic migraine	ICHD-3	Single (proximal) US-guided GONB	Repeated, PRF (GON)	Pain intensity and frequency, and headache frequency	3 ml bupivacaine 0.5%, and 2 cc saline solution	1 month
Turan 2023 [29]	Turkey	Non-RCT	60	Chronic migraine with cutaneous allodynia	ICHD-3	Repeated (proximal) US-guided GONB	Repeated (proximal) US-guided GONB, and TPI with or without PTNB	NRS, HIT-6, brush allodynia test, and number of analgesic tablets	1–5 mL of 0.5% bupivacaine administered to each block	3 months

*EHF* European Headache Federation, *GONB* Greater Occipital Nerve Block, *HIT-6* Headache Impact Test-6, *ICHD* International Classification of Headache Disorders, *MIDAS* Migraine Disability Assessment Scale, *NA* Not Applicable, *NRS* Numerical Rating Scale, *PRF* Pulsed Radiofrequency, *PTNB* Peripheral Trigeminal Nerve Block, *RCT* Randomized Controlled Trial, *SPG* Sphenopalatine Ganglion, *TPI* Trigger Point Injection, *US* Ultrasound, *VAS* Visual Analog Scale

**Table 2:** Baseline of the included studies

Study ID	Sample size			Males, n (%)			Age, Mean (SD)			Migraine prophylaxis drugs, n (%)			Groups names		
	G 1	G 2	G 3	G 1	G 2	G 3	G 1	G 2	G 3	G 1	G 2	G 3	G 1	G 2	G 3
Taha 2025 [25]	21	22	10	1 (4.76)	3 (13.64)	1 (10)	30.62 (11.28)	37.32 (10.74)	35.2 (8.8)	NA	NA	NA	Repeated (proximal) US-guided GONB	Repeated SPG	Sham
Saracoglu 2024 [26]	16	16	NA	3 (18.75)	2 (12.5)	NA	37.56 (9.67)	35.88 (9)	NA	NA	NA	NA	Single (distal) US-guided GONB	Single PRF + GONB	NA
Ertilav 2024 [28]	35	32	NA	10 (28.6)	2 (6.3)	NA	41.3 (12.6)	44.8 (9.6)	NA	NA	NA	NA	Repeated (distal) US-guided GONB	Single PRF	NA
Balta 2023 [30]	37	33	NA	NA	NA	NA	25.75 (11.76)	27.67 (13.17)	NA	NA	NA	NA	Repeated (proximal) US-guided GONB	Repeated SPG	NA
Perdecioglu 2023 [27]	35	27	NA	8 (61.5)	5 (38.5)	NA	42.11 (10.69)	42.3 (9.16)	NA	NA	NA		Single (proximal) US-guided GONB	Repeated PRF	NA
Turan 2023 [29]	20	18	22	7 (35)	8 (44.4)	5 (22.7)	44.15 (10.973)	47.33 (10.43)	39 (8.379)	Topiramate: 6 (30%); sodium valproate: 6 (30%); SNRIs: 5 (25%); amitriptyline: 4 (20%); flunarizine: 2 (10%); propranolol: 3 (15%)	Topiramate: 3 (16.7%); sodium valproate: 5 (27.8%); SNRIs: 6 (33.3%); amitriptyline: 5 (37.8%); flunarizine: 2 (11.1%); propranolol: 0 (0%)	Topiramate: 7 (31.8%); sodium valproate: 8 (36.4%); SNRIs: 6 (27.3%); amitriptyline: 3 (13.6%); flunarizine: 1 (4.5%); propranolol: 3 (13.6%)	Repeated (proximal) US-guided GONB	Repeated (proximal) US-guided GONB + TPI	Repeated (proximal) US-guided GONB + TPI and PTNB

*GONB* Greater Occipital Nerve Block, *NRS* Numerical Rating Scale, *PRF* Pulsed Radiofrequency, *PTNB* Peripheral Trigeminal Nerve Block, *RCT* Randomized Controlled Trial, *SD* Standard Deviation, *SNRIs* Serotonin-Norepinephrine Reuptake Inhibitors, *SPG* Sphenopalatine Ganglion, *TPI* Trigger Point Injection, *US* Ultrasound

### Bias risk assessment

Among the four RCTs assessed using the ROB-2 tool, two were rated as high risk of bias [25, 28], one as low risk [26], and one as having some concerns [27] (Supplementary Table 1). One observational study evaluated with NOS was considered low risk [30] (Supplementary Table 2). In comparison, one non-RCT study assessed with the ROBINS-I tool was rated as a serious risk of bias [29] (Supplementary Table 3).

### Pain intensity at one month

The pain intensity was defined by visual analog scale (VAS) from 0-10 cm or numeric rating scale (NRS) from 0-10. The analysis included four studies with 107 patients in the US-GONB group and 107 in the control group. A subgroup analysis was done based on the control group.

#### US-GONB vs. PRF

**Fig. 2:**
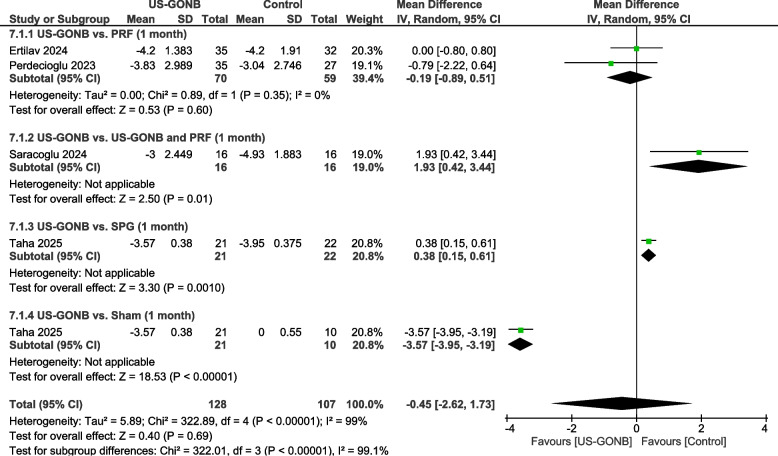
Pain intensity at one month. Meta-analysis of pain intensity at one month among patients treated with US-GONB. Compared with PRF, no significant difference was found (MD: −0.19 points, 95% CI: [−0.89, 0.51], *P* = 0.6). US-GONB combined with PRF significantly reduced pain compared to US-GONB alone (MD: 1.93 points, 95% CI: [0.42, 3.44], *P* = 0.01). The analysis favored SPG compared to US-GONB (MD: 0.38 points, 95% CI: [0.15, 0.61], *P* = 0.001). A significant difference was also observed between US-GONB and sham, favoring US-GONB (MD: −3.57 points, 95% CI: [−3.95, −3.19], *P* < 0.00001)

This subgroup included two studies with 70 patients in the GONB group and 59 patients in the PRF group. The analysis revealed no statistically significant difference between the groups (MD: −0.19 points, 95% CI: [−0.89, 0.51], *P* = 0.6). No significant heterogeneity existed between studies (*I*^2^ = 0%, *P* = 0.35) (Fig. 2).

#### US-GONB vs. US-GONB and PRF

This subgroup included one study involving 16 patients in the US-GONB group and 16 in the US-GONB and PRF group. The pooled analysis favored the US-GONB and PRF group (MD: 1.93 points, 95% CI: [0.42, 3.44], *P* = 0.01) (Fig. 2).

#### US-GONB vs. SPG

This subgroup included one study with 21 patients in the US-GONB group and 22 in the SPG group. The pooled analysis favored the SPG group (MD: 0.38 points, 95% CI: [0.15, 0.61], *P* = 0.001) (Fig. 2).

#### US-GONB vs. sham

This subgroup included one study with 21 patients in the US-GONB group and 10 in the sham group. The comparison with the sham group significantly favored US-GONB (MD: −3.57 points, 95% CI: [−3.95, −3.19], *P* < 0.00001) (Fig. 2).

### Monthly headache days at one month

The analysis included four studies with 109 patients in the US-GONB group and 108 in the control group. A subgroup analysis was done based on the control group.

#### US-GONB vs. PRF

**Fig. 3:**
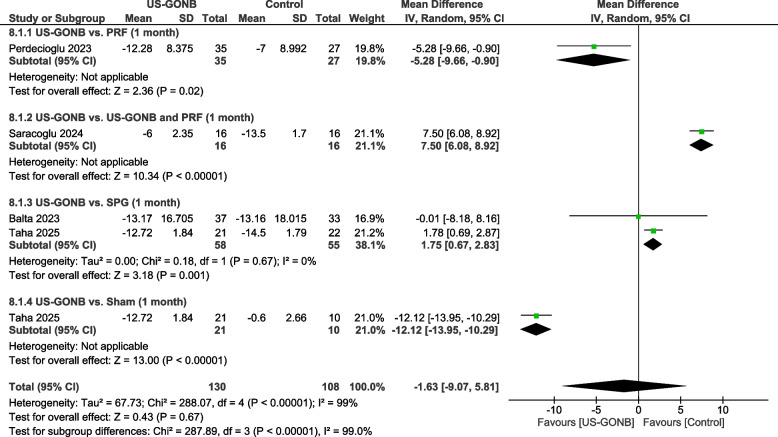
Monthly headache days at one month. Meta-analysis of headache frequency (days per month) at one month among patients treated with US-GONB. Compared to PRF, the results favored US-GONB (MD: −5.28 days, 95% CI: [−9.66, −0.9], *P* = 0.02). US-GONB combined with PRF showed a significant reduction in headache frequency when compared to US-GONB alone (MD: 7.5 days, 95% CI: [6.08, 8.92], *P* < 0.00001). The analysis favored SPG compared to US-GONB (MD: 1.75 days, 95% CI: [0.67, 2.83], *P* = 0.001). A significant difference was also observed between US-GONB and sham, favoring US-GONB (MD: −12.12, 95% CI: [−13.95, −10.29], *P* < 0.00001)

This subgroup included a single study with 35 patients in the US-GONB group and 27 in the PRF group. The results favored the US-GONB group (MD: −5.28 days, 95% CI: [−9.66, −0.9], *P* = 0.02) (Fig. 3).

#### US-GONB vs. US-GONB and PRF

This subgroup included a single study with 16 patients in the US-GONB group and 16 in the US-GONB and PRF one. The results favored the US-GONB combined with the PRF group (MD: 7.5 days, 95% CI: [6.08, 8.92], *P* < 0.00001) (Fig. 3).

#### US-GONB vs. SPG

This subgroup included two studies involving 58 patients in the US-GONB group and 55 in the SPG group. The analysis revealed a statistically significant difference between the groups, favoring the SPG group (MD = 1.75 days, 95% CI: [0.67, 2.83], *P* = 0.001). No significant heterogeneity existed between studies (*I*^2^ = 0%, *P* = 0.67) (Fig. 3).

#### US-GONB vs. sham

This subgroup included one study with 21 patients in the US-GONB group and 10 in the sham group. The results favored the US-GONB group (MD: −12.12 days, 95% CI: [−13.95, −10.29], *P* < 0.00001) (Fig. 3).

### Headache attack duration (hours) at one month

The analysis included two studies with 58 patients in the US-GONB group and 65 in the control group. A subgroup analysis was done based on the control group.

#### US-GONB vs. SPG

**Fig. 4:**
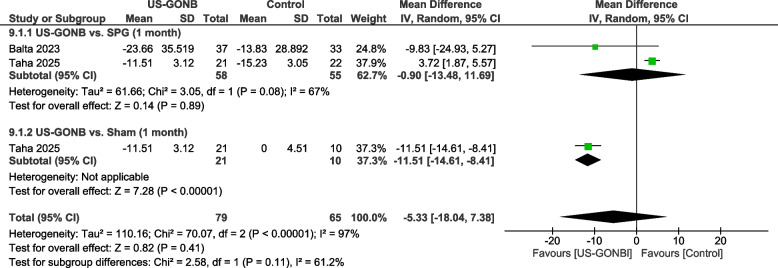
Headache attack duration (hours) at one month. Meta-analysis of headache attack duration (hours) at one month among patients treated with US-GONB. No significant difference was observed between US-GONB and SPG (MD: −0.9 hours, 95% CI: [−13.48, 11.69], P = 0.89). An important difference was found between US-GONB and sham, favoring US-GONB (MD: −11.51 hours, 95% CI: [−14.61, −8.41], P < 0.00001)

This subgroup included two studies involving 58 patients in the US-GONB group and 55 in the SPG group. The analysis revealed no statistically significant difference between the groups (MD = −0.9, 95% CI: [−13.48, 11.69], *P* = 0.89). The results were heterogeneous (*I*^2^ = 67%, *P* = 0.08) (Fig. 4).

#### US-GONB vs. sham

This subgroup included one study with 21 patients in the US-GONB group and 10 in the sham group. The comparison significantly favored the US-GONB group (MD: −11.51 hours, 95% CI: [−14.61, −8.41], *P* < 0.00001) (Fig. 4).

### Monthly number of analgesic drugs taken at one month

The analysis included three studies with 57 patients in the US-GONB group and 88 in the control group. A subgroup analysis was done based on the control group.

#### US-GONB vs. US-GONB and PRF

**Fig. 5:**
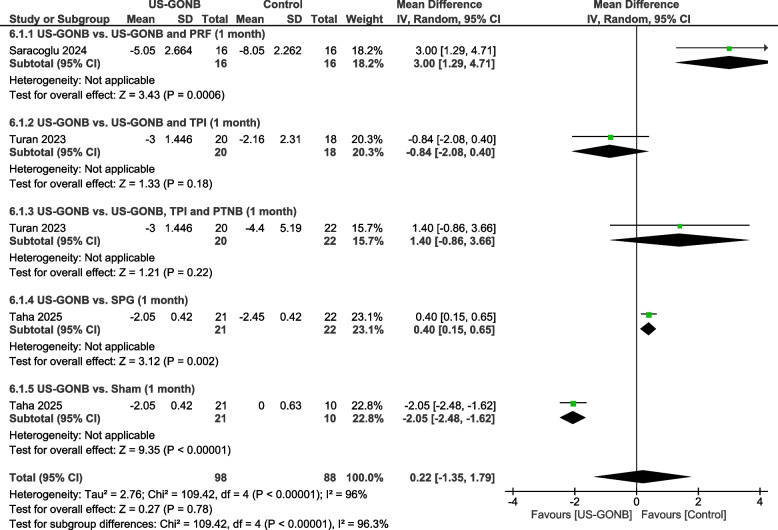
Monthly number of analgesic drugs taken at one month. Meta-analysis of the monthly number of analgesic drugs taken at one month among patients treated with US-GONB. The comparison favored US-GONB combined with PRF when compared to US-GONB alone (MD: 3 analgesics, 95% CI: [1.29, 4.71], *P* = 0.0006). No significant difference was found between US-GONB and US-GONB combined with TPI (MD: −0.84 analgesics, 95% CI: [−2.08, 0.4], *P* = 0.18), or between US-GONB and US-GONB combined with both TPI and PTNB (MD: 1.4 analgesics, 95% CI: [−0.86, 3.66], *P* = 0.22). The analysis favored SPG compared to US-GONB (MD: 0.4 analgesics, 95% CI: [0.15, 0.65], *P* = 0.002). A significant difference was observed between US-GONB and sham, favoring US-GONB (MD: −2.05 analgesics, 95% CI: [–2.48, −1.62], *P* < 0.00001)

This subgroup included one study with 16 patients in the US-GONB group and 16 in the US-GONB plus PRF one. The comparison significantly favored the US-GONB combined with the PRF group (MD: 3 analgesics, 95% CI: [1.29, 4.71], *P* = 0.0006) (Fig. 5).

#### US-GONB vs. US-GONB and TPI

This subgroup included one study with 20 patients in the US-GONB group and 18 in the US-GONB combined with the TPI group. The findings showed no significant difference between groups (MD: −0.84 analgesics, 95% CI: [−2.08, 0.4], *P* = 0.18) (Fig. 5).

#### US-GONB vs. US-GONB, TPI, and peripheral trigeminal nerve block (PTNB)

This subgroup included one study comparing 20 patients in the US-GONB group with 22 in the US-GONB combined with the TPI and PTNB group. No statistically significant difference was observed between the groups (MD: 1.4 analgesics, 95% CI: [−0.86, 3.66], *P* = 0.22) (Fig. 5).

#### US-GONB vs. SPG

This subgroup included one study with 21 patients in the US-GONB group and 22 in the SPG group. The analysis favored the SPG group (MD: 0.4 analgesics, 95% CI: [0.15, 0.65], *P* = 0.002) (Fig. 5).

#### US-GONB vs. sham

This subgroup included one study comparing 21 patients in the US-GONB group with 10 in the sham group. The analysis significantly favored the US-GONB group (MD: −2.05 analgesics, 95% CI: [–2.48, −1.62], *P* < 0.00001) (Fig. 5).

### Adverse events (AEs)

The included studies reported multiple AEs associated with US-GONB. The most frequently observed AE was dizziness, occurring in 38.9% of patients who received US-GONB (46/118), while it was not reported in the control groups (SPG: 0/55; sham: 0/10). Moderate-to-severe migraine attacks were observed in 29.7% of patients in the US-GONB group (11/37), compared to 21.2% in the SPG group (7/33). Vertigo occurred in 2.1% of US-GONB patients (1/37) and was not reported in the SPG group. Pain at the injection site was reported in 5.2% of US-GONB patients (3/58), compared to 16.4% in the SPG group (9/55) and none in the sham group (0/10). Bleeding at the injection site was not observed in the US-GONB group (0/58), whereas it occurred in 45.5% of the SPG group (25/55) and was absent in the sham group (0/10). Notably, lacrimation was reported in 87.9% of the SPG group (29/33) but was not observed in the US-GONB group (0/37). For further details, see Supplementary Table 4.

## Discussion

Our first systematic review and meta-analysis included six eligible studies encompassing 344 participants. Relative to sham injections, US-GONB has shown significant, clinically meaningful reductions in pain intensity (MD = –3.6 points), monthly headache days (around 12 day per month), headache attack duration, and monthly analgesic use. Efficacy versus active comparators was mixed: outcomes were comparable to PRF alone, superior when US-GONB was combined with PRF, and generally inferior to SPG block in direct head-to-head trials. Safety signals were reassuring. Dizziness was the most common adverse event (38.9%), while serious complications such as bleeding were rare and occurred primarily in comparator groups.

Interventional approaches in our included studies cluster into two broad strategies: repeated or single US-GONB, delivered either proximally (most trials) or distally (Saracoglu 2024, Ertilav 2024), versus comparators. In a double-blind RCT of 50 adults with post-dural-puncture headache, US-GONB given proximally at the C2 level appears to provide quicker and more durable relief than the distal approach. At 24 h, clinical success (sitting NRS < 4) reached 84% with the proximal block versus 60% distally, and cumulative 48‑hour consumption of paracetamol and tramadol was significantly less in the proximal group [31]. Other trials suggest the superiority of proximal over distal PRF of the GONB in migraine management [32]. However, the state of evidence remains inconclusive.

The evidence found for US-GONB was supported multi-dimensionally across multiple measures of efficacy. A decrease of 12 days compared to sham stimulation appears to be well over the regulatory guidance that defines a clinically meaningful response as a 50% decrease in monthly migraine days from the baseline, as a minimally clinically significant difference (MCID) [33]. Similarly, the difference of 3.6 points in pain intensity is higher than the 3-points MCID in the literature [34]. While other comparisons in monthly headache days were statistically significant, they may be less clinically significant. This was also reflected in different outcomes, where the comparison between US-GONB and PRF was not substantial for pain intensity. In contrast, the comparison between US-GONB and the combination of US-GONB and PRF was significant for all other outcomes, suggesting a possible synergic effect. The comparison of US-GONB across outcomes was either small, significant, or insignificant, which may support the robustness of US-GONB compared to other established treatments.

Balta et al.conducted a retrospective two-center cohort study of 70 adults with chronic migraine, comparing four weekly (US-GONB, *n* = 37) with (SPG, *n* = 33). By month 3, both interventions produced a median 80% reduction in monthly headache days from baseline (median percentage change 81% for US-GONB and 85% for SPG, comfortably exceeding the ≥ 50% MCID). Responder rates (≥ 50% reduction) were similar at 76% versus 73%. Pain intensity fell by roughly one-third in both groups, surpassing the 30 mm VAS MCID, while baseline medication-overuse prevalence plummeted from 78 to 13% with US-GONB and 58% to 9% with SPG. No serious adverse events were reported, only mild transient effects such as local pain or lacrimation. However, it was a retrospective study, and RCTs further supported their findings.

Taha et al. randomized 53 adults with chronic, treatment-resistant migraines to a single ultrasound-guided SPG, a US-GONB, or a sham saline injection. One-month outcomes showed large within-group improvements for both active arms versus sham: monthly headache days were roughly halved with SPG (28 → 13.7; –14.5 days, –51%) and cut by 45% with US-GONB (28 → 15.7); attack duration fell by 54% and 51%, respectively, and pain intensity dropped 4 points on a 0–10 NRS in both groups. Total pain burden plunged 83% with SPG and 73% with US-GONB, while daily analgesic use fell by 60%, and analgesic-use days per month declined by two-thirds. Disability improved markedly: headache impact test-6 (HIT-6) dropped 18 points, and migraine disability assessment scale (MIDAS) scores fell 46–70 days at three months (more than the 5-point MCID). Although overall efficacy did not differ significantly between SPG and US-GONB, SPG achieved a 100% responder rate among patients with cranial autonomic symptoms versus 47% with US-GONB (*p* = 0.020). Both procedures were well tolerated, with transient local pain or minor bleeding.

Perdecioglu et al. conducted a single-blinded RCT that enrolled 62 adults with chronic migraine to receive either two sessions of PRF applied along the greater-occipital-nerve trace or a single US-GONB. After four weeks, mean pain intensity on a 0–10 VAS fell from 8.78 to 5.74 in the PRF arm (−3.0 points; −34%) and from 8.14 to 4.31 in the US-GONB arm (−3.8 points; −47%), with no significant between-group difference (*p* = 0.070); both reductions exceeded 2 points on pain scores. Monthly headache days decreased from 19 to 12 with PRF (−44%) and from 22 to 9 with US-GONB (−57%); only the latter crossed the customary 50% responder threshold, yet the groups remained statistically similar (p = 0.170). Adverse events were mild: transient neck erythema in six PRF patients and a single hypotension-with-hyperalgesia episode after injection in the US-GONB group, with no serious complications reported.

Similarly, Ertilav et al. randomized 67 adults with refractory chronic migraine to either four weekly (US-GONB, *n* = 35) or a single 240-s PRF session to the same nerve (PRF, *n* = 32). Both arms achieved significant early pain reductions, meaning VAS fell by 4.2 points in one month (54% from 7.7 to 8.0), easily exceeding the MCID. At six months, PRF sustained a 4.8-point drop (3.2 ± 1.2), whereas the block arm rebounded slightly to a 3.7-point drop (4.0 ± 1.4), yielding a clinically relevant between-group advantage of −0.8 points for PRF (*p* = 0.009). Disability followed the same pattern: MIDAS grade improved by 1.4 categories in both groups at one month (from severe Grade III–IV to mild–moderate); by six months, PRF maintained a 1.6-grade gain (mean 1.3), whereas US-GONB regressed to a 1.1-grade gain (mean 2.0), with PRF superior (*p* < 0.001). Analgesic-withdrawal rates were comparable: about 60% of participants reduced acute medication use at one month, and differences remained non-significant at six months (*P* = 0.06).

Saracoglu et al. explored the synergic effect of combining the two modalities in a double-blind, two-arm RCT in 32 adults with chronic migraine, in which every participant received US-GONB; half were then randomized to an additional 4-min PRF (GONB + PRF). By month 6, mean pain intensity on the 0–10 VAS had fallen from 8.63 to 3.50 in the US-GONB + PRF arm (−5.1 points, −60%) versus 8.75 to 6.81 with US-GONB alone (−1.9 points, −22%); the between-group gap of − 3.3 points met significance (*p* = 0.002). Monthly migraine attacks decreased 84% (9.6 → 1.56) with PRF but only 15% (9.4 → 8.0) with block alone, a difference that crossed both the 50% responder threshold and statistical significance at every follow-up (*p* < 0.001) [35]. Analgesic consumption mirrored these patterns, dropping 84% (9.95 → 1.63) with PRF against a 27% fall (10.25 → 7.44) with US-GONB alone (*p* = 0.002), again a clinically meaningful reduction.

Turan et al. conducted a comparative study in 60 chronic migraine patients with cutaneous allodynia, who received four weekly sessions of proximal US-GONB, US-GONB plus TPI, or GONB + TPI + PTNB. In the most intensive arm (GONB + TPI + PTNB), headache-related disability (HIT-6) dropped by 16 points (76 → 60); pain intensity (NRS) improved by 6 points (10 → 4); and monthly migraine days declined by about 40% (10 → 6). Median HIT-6 scores fell from 78 to 63 in the GONB arm (−15 points, 19%) and from 76 to 62.5 with GONB + TPI (−13.5 points, 18%), with all regimens achieving well above the 5–6 point MCID change from baseline, and the triple therapy achieving a higher MCID of 2.3 compared to US-GONB alone [36, 37]. The triple regimen yielded significantly lower disability at 3 months than the other strategies (*p* < 0.001). Adverse events were limited to transient dizziness or nausea in 18 participants, resolving within 15 min.

Other interventions have also been tested for efficacy in migraine. A recent study evaluated the use of combination therapy with botulinum toxin A (BTA) injections followed by PRF of the GONB, which resulted in patients being free of adverse events and experiencing a marked reduction in pain intensity at one month [38]. These findings suggest that combined therapy may be a safe and effective option for migraine management. Furthermore, a recent meta-analysis reported that GONB significantly reduced pain intensity in both migraine and tension-type headache patients [39].

Despite this being the first systematic review to assess the evidence on US-GONB, our study had several limitations. The evidence base is limited by a small number of studies, sample sizes, and heterogeneous comparators, which limited our ability to conduct a meta-analysis, with most evidence in each comparison arising from 1 or 2 studies. However, we performed quantitative and qualitative evidence syntheses to view its state comprehensively. In addition, the generalizability of our findings may be limited, as most of the included studies were conducted in Turkey, with only one study in Egypt. We also included both RCTs and observational studies; however, they all reported the same effect measures, allowing for their inclusion in the analysis [40]. 

The present synthesis suggests that US-GONB may represent an effective and well-tolerated option when landmark-guided blocks or pharmacotherapy fail and highlights potential synergism when combined with PRF. However, the degree of current evidence prevents us from acquiring detailed information on its efficacy compared to other interventions. Future research should prioritize adequately powered, multicenter randomized trials that compare standardized US-GONB protocols with other image-guided nerve or ganglion interventions, clarify the additive value of PRF, and incorporate health-economic analyses to guide resource allocation. Harmonizing outcome definitions and reporting will also facilitate more precise evidence synthesis and network meta-analyses as we advance.

## Conclusion

This systematic review and meta‑analysis suggest that US‑GONB may confer meaningful benefits for chronic migraine. Compared with sham injection, US‑GONB markedly reduced monthly headache days, pain intensity, attack duration, and analgesic consumption. Efficacy relative to active comparators was heterogeneous: outcomes were comparable to PRF alone, superior when PRF was added to US‑GONB, and generally inferior to SPG in direct head‑to‑head trials. The procedure was well tolerated; dizziness was the most frequent adverse event, and serious complications were rare. Collectively, these findings indicate that US-GONB may be a safe, minimally invasive therapeutic option either as a standalone or as part of a combined neuromodulation strategy with PRF. Nevertheless, the evidence is constrained by small sample sizes, varied protocols (proximal vs. distal approaches, single vs. repeated sessions), and a moderate‑to‑high risk of bias in several studies. Future work should prioritize adequately powered, multicenter randomized trials employing standardized US‑GONB techniques, exploring protocols, anatomical approaches, and additive effects of PRF or other blocks, while incorporating cost‑effectiveness and long‑term safety outcomes, before fully integrating into guideline‑based migraine management plans.

## Supplementary Information

Supplementary Material 1.

## Data Availability

This systematic review and meta-analysis relied on publicly available data from previously published studies.
