# Supplementary material for: Ultrasound-guided greater occipital nerve block for chronic migraine: a systematic review and meta-analysis
Source: Head Face Med. 2025 Nov 24;21:81. doi: 10.1186/s13005-025-00554-1 (PMC12702149; doi:10.1186/s13005-025-00554-1)
Supplement: Supplementary file 1 — Supplementary Material 1. [file 13005_2025_554_MOESM1_ESM.docx]

**Supplementary Table 1. Risk of bias assessment of Randomized Clinical trials using Cochrane Risk of Bias 2.0 (RoB 2)**

| **Study ID** | **D1** | **D2** | **D3** | **D4** | **D5** | **Overall** |
| --- | --- | --- | --- | --- | --- | --- |
| Taha 2025 | Some concerns | Some concerns | Some concerns | Some concerns | Some concerns | High risk |
| Saracoglu 2024 | Low | Low | Low | Low | Low | Low risk |
| Ertilav 2024 | Some concerns | Some concerns | Low | Some concerns | Low | High risk |
| Perdecioglu 2023 | Some Concerns | Low | Some Concerns | Low | Low | Some Concerns |

**D1:** Bias arising from the randomization process; **D2:** Bias due to deviations from intended interventions; **D3:** Bias due to missing outcome data; **D4:** Bias in measurement of the outcome; **D5:** Bias in selection of the reported result.

**Supplementary Table 2. Risk of bias assessment of observational studies using Newcastle-Ottawa Scale (NOS)**

| **Study** | **Selection** | | | | **Comparability** | **Outcome** | | | **Quality** |
| --- | --- | --- | --- | --- | --- | --- | --- | --- | --- |
|  | Representativeness of the exposed cohort | Selection of the non-exposed cohort | Ascertainment of exposure | The outcome of interest was not present at start of study |  | Assessment of outcome | Was follow-up long enough for outcomes to occur | Adequacy of follow up of cohorts |  |
| Balta 2023 | ⭐ | ⭐ | ⭐ | ⭐ | ⭐ | ⭐ | ⭐ | ⭐ | 8/9 (Good) |

**Supplementary Table 3. Risk of bias assessment of non-randomized studies using ROBINS-1**

| **Study** | **D1** | **D2** | **D3** | **D4** | **D5** | **D6** | **D7** | **Overall** |
| --- | --- | --- | --- | --- | --- | --- | --- | --- |
| Turan 2023 | Serious | Moderate | Moderate | Moderate | Moderate | Low | Moderate | Serious |

**D1** Bias due to confounding; **D2** Bias in the selection of participants into the study; **D3** Bias in the classification of interventions; **D4** Bias due to deviations from intended interventions, **D5** Bias due to missing data; **D6** Bias in the measurement of outcomes; **D7** Bias in the measurement of outcomes

**Supplementary Table 4. Summary of Adverse Events in US-guided GONB and Control Groups**

| Adverse events | Location of GONB | Studies reported | The incidence rate in the US-guided GONB group (Event/Total, (%)) | The incidence rate in the control group (Group Name: Event/Total, (%)) |
| --- | --- | --- | --- | --- |
| Dizziness | Proximal | 2 | 46/118, (38.9%) | SPG: 0/55, (0.0%), sham: 0/10, (0.0%) |
| Moderate-to-severe migraine attack | Proximal | 1 | 11/37, (29.7%) | SPG: 7/33, (21.2%) |
| Vertigo | Proximal | 1 | 1/37, (2.1%) | SPG: 0/33, (0.0%) |
| Lacrimation | Proximal | 1 | 0/37, (0.0%) | SPG: 29/33, (87.9%) |
| Pain at the site of injection | Proximal | 2 | 3/58, (5.2%) | SPG: 9/55, (16.4%), sham: 0/10, (0.0%) |
| Bleeding at the site of injection | Proximal | 2 | 0/58, (0.0%) | SPG: 25/55, (45.5%), sham: 0/10, (0.0%) |

GONB: greater occipital nerve block; SPG: sphenopalatine ganglion; US: ultrasound
